# Graphene as a Buffer Layer for Silicon Carbide-on-Insulator Structures

**DOI:** 10.3390/ma5112270

**Published:** 2012-11-09

**Authors:** Budi Astuti, Masahiro Tanikawa, Shaharin Fadzli Abd Rahman, Kanji Yasui, Abdul Manaf Hashim

**Affiliations:** 1 Ibnu Sina Institute for Fundamental Science Studies, Faculty of Electrical Engineering, Universiti Teknologi Malaysia, Skudai, Johor 81310, Malaysia; E-Mails: b_astuti79@yahoo.com (B.A.); shaharinfadzli@fke.utm.my (S.F.A.R.); 2 Department of Physics, Faculty of Mathematics and Nature Science, Semarang State University, Semarang, Jawa Tengah 50229, Indonesia; 3 Department of Electrical Engineering, Nagaoka University of Technology, Kamitomioka, Nagaoka, Niigata 9402136, Japan; E-Mails: tanikawa@stn.nagaokaut.ac.jp (M.T.); kyasui@vos.nagaokaut.ac.jp (K.Y.); 4 Malaysia-Japan International Institute of Technology, Universiti Teknologi Malaysia, Jalan Semarak, Kuala Lumpur 54100, Malaysia

**Keywords:** graphene, silicon carbide, insulator, buffer layer, hot-mesh CVD

## Abstract

We report an innovative technique for growing the silicon carbide-on-insulator (SiCOI) structure by utilizing polycrystalline single layer graphene (SLG) as a buffer layer. The epitaxial growth was carried out using a hot-mesh chemical vapor deposition (HM-CVD) technique. Cubic SiC (3C-SiC) thin film in (111) domain was realized at relatively low substrate temperature of 750 °C. 3C-SiC energy bandgap of 2.2 eV was confirmed. The Si-O absorption band observed in the grown film can be caused by the out-diffusion of the oxygen atom from SiO_2_ substrate or oxygen doping during the cleaning process. Further experimental works by optimizing the cleaning process, growth parameters of the present growth method, or by using other growth methods, as well, are expected to realize a high quality SiCOI structure, thereby opening up the way for a breakthrough in the development of advanced ULSIs with multifunctionalities.

## 1. Introduction

The performance of silicon large-scale integrated circuits (Si-LSIs) has been enhanced over the last 30 years by increasing the number of transistors in accordance with Moore’s law [1]. The transistor number in the latest processor of ultra-LSIs (ULSIs) is already over one billion [1]. The scaling rule of the Si transistor has made it possible to enhance the performance of the ULSIs. However, the miniaturization of the transistors becomes increasingly difficult owing to the physical limitations, and the conventional scaling rule will not be enough to enhance the performance of the LSIs. Recently, the concept of advanced heterogeneous integration on Si platform was proposed by Takagi *et al.* towards the realization of a so-called “More than Moore” technology [2]. They proposed new semiconductor materials with higher mobility than Si such as germanium (Ge) and III-V (*i.e.*, gallium arsenide, GaAs) materials to be introduced on the Si platform in order to not only enhance the performance of MOS transistors [3] but also to facilitate the present ULSIs with various functionalities where these materials can be used to fabricate various kinds of functional devices, such as optical devices [4], photodetectors [5] and solar batteries [6].

In addition to Ge and III-V materials as proposed by them, we believe that graphene and IV-IV materials *(i.e.*, silicon carbide, SiC) are also the promising materials for such purposes due to their superior properties. Graphene, a carbon allotrope, possesses high carrier mobility, up to 200,000 cm^2^/Vs, even at room temperature (RT) [7], and this mobility, in turn, results in a long mean free path of 1.2 µm at a carrier concentration of 2 × 10^11^ cm^−2^. The quantum Hall effect exists in graphene at RT, owing to ballistic transport of electrons and holes [8], and this means graphene is potentially useful for ballistic device applications. Graphene has also been shown as a material with high thermal conductivity [9,10,11,12]. SiC, a wide bandgap semiconductor, is expected to be used in electronics devices for high-frequency, high-power, high-temperature applications and also valuable for fabricating microelectromechanical systems (MEMS) that can be operated in harsh environments [13]. As a next-generation technology, such intelligent system-on-chip (i-SoC) on silicon is considered as a promising and practical direction. In order to be able to fabricate electronic devices in those materials, it is necessary to electronically isolate these materials and the Si substrate by insulator. Therefore, some breakthrough on growth technologies are strongly required to realize high quality Ge-on-insulator (GOI) [14], graphene-on-insulator [15], GaAs-on-insulator [16] and SiC-on-insulator (SiCOI) [17] structures.

So far, to realize high quality SiC thin film on silicon, we have studied the growth of SiC-on-SOI substrates with an ultrathin top Si layer [18] and also the introduction of a carbonization layer prior to SiC growth on Si substrate [19]. The latter work triggers an idea to employ graphene, a two-dimensional carbon material with only one atomic layer as a buffer layer for the epitaxial growth of SiC on insulator. An additional motivation for the present study is because both graphene and SiC are very good heat conductors and thus, a major issue of thermal management in heterogeneous integration can also be solved. The attempt to grow SiCOI by introducing a graphene buffer layer has not yet been reported. In this pioneer work, we reported the feasibility to grow 3C-SiC thin film on graphene/SiO_2_/Si substrate at relatively low temperature by a homemade hot-mesh chemical vapor deposition (HM-CVD) apparatus. There are several reports regarding to the growth of SiC film on SiO_2_ substrates without introduction of any buffer layer [20,21]. In those reports, the processes were done by different kinds of techniques such as low-pressure CVD (LPCVD) and atmospheric-pressure CVD (APCVD). The source gases were silane and propane, and the growth temperatures were above 1000 °C. Previously, our group had also conducted the experimental growth of 3C-SiC film on the SiO_2_ substrate without a graphene buffer layer using the same procedures with this work in terms of source gas, growth temperature, and technique [22]. We reported that at growth temperature of 750 °C, only the diffraction peak of 3C-SiC (200) was clearly observed. However, since SiO_2_ is amorphous, all reported grown SiC films, including those from our work, show polycrystalline structures.

Based on the above results, one of major merits of using graphene as a buffer layer is the feasibility of growing highly oriented single crystalline SiC film. Large lattice mismatches between graphene and SiC is a critical problem in realizing high quality epitaxial film, but we believe such a problem can be resolved by clever growth technique. As an example, it was proved that the large density of threading dislocations resulted in the growth of GaN on sapphire due to a large lattice mismatch has been drastically reduced by the application of lateral growth technique [23]. Graphene is a two-dimensional hexagonal network of carbon atoms which is formed by making strong triangular σ-bonds of the sp^2^ hybridized orbitals. This bonding structure is similar to the C plane of a hexagonal crystalline structure and (111) plane of zinc-blende structure. With this regard, the growth of (111) oriented SiC on graphene in <111> direction is feasible. Recently, graphene was also shown to be able to serve as a buffer layer for the growth of other material nanostructures on insulator such as zinc oxide (ZnO) nanorods [24] due to the similarity in bonding structures.

## 2. Experimental

**Figure 1:**
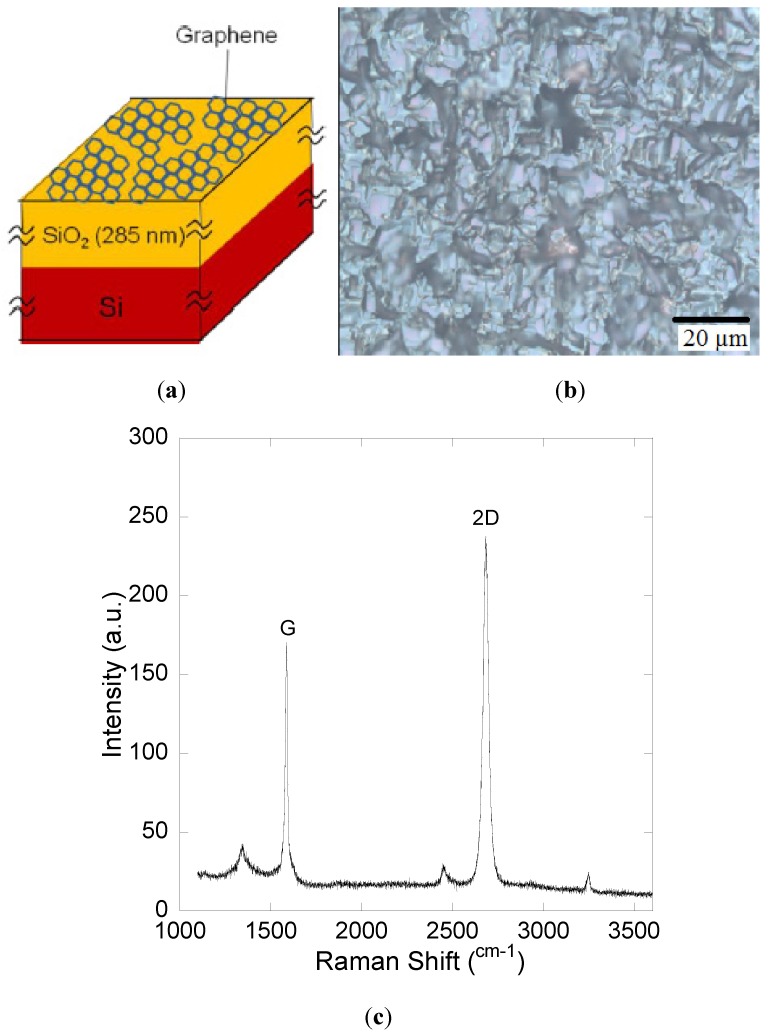
(**a**) Schematic of substrate (bird’s eye view); (**b**) Nomarski photo of graphene grains (top view) and (**c**) Raman spectra of CVD grown graphene grains.

The schematic of graphene/SiO_2_/Si(100) substrate, purchased from Graphene Laboratories Inc., NY, USA, is shown in Figure 1a. As shown in Figure 1b, the surface of substrate consists of many graphene grains with the maximum diameter up to 20 µm. The graphene grains were grown by CVD with the coverage of single layer graphene (SLG) grains of 90%. The thickness of graphene grains was also confirmed by the Raman measurement (Ar^+^ laser at incident power of 20 mW/wavelength of 514.5 nm), as shown in Figure 1c.

The sharp peaks of the G and 2D band were observed at 1580 cm^−1^ and 2670 cm^−1^, respectively. The ratio intensity of the 2D and G band (I_2D_/I_G_) is about 1.6, hence it indicates that the graphene is a single layer [25]. In general, the 2D band spectra change its shape, width, and position with the increase of layer number. At that time, the G band peak position will also change by downshifting to a lower wave number due to the chemical bonding between the graphene layer [26]. Traditional organic cleaning was applied prior to the growth which consists of acetone, ethanol and deionized (DI) water.

**Figure 2:**
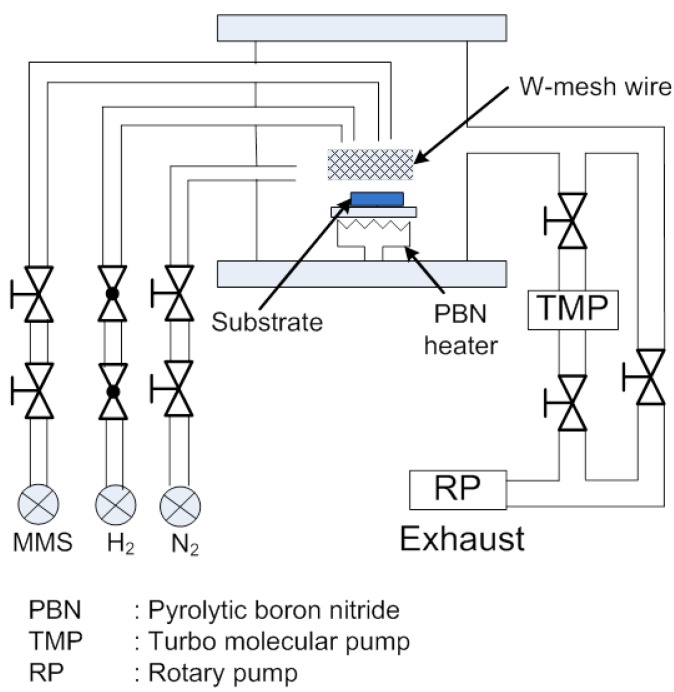
Schematic of a homemade hot-mesh CVD apparatus.

Figure 2 shows the schematic of a homemade hot-mesh CVD apparatus. The distance between the tungsten mesh (wire diameter of 0.1 mm, 30 mesh/in.) and the substrate was set to 30 mm. Monomethylsilane (MMS) was used as a single source precursor and H_2_ as a carrier gas with the constant flow rate of 100 sccm. This method utilizes heated tungsten wires arranged in a mesh, which promotes the high decomposition efficiency of H_2_ gas. The growth pressure was set to 1.8 Torr. The temperature of the substrate was set to 750 °C, while the mesh was 1800 °C. As reported, among its many polytypes, 3C-SiC is a stable phase that can be grown at low temperatures [27]. The crystallinity, orientation and chemical bonding of the grown film were characterized using X-Ray diffraction (XRD), photoluminescence (PL) and Fourier transforms infrared (FTIR) measurement.

## 3. Results and Discussion

**Figure 3:**
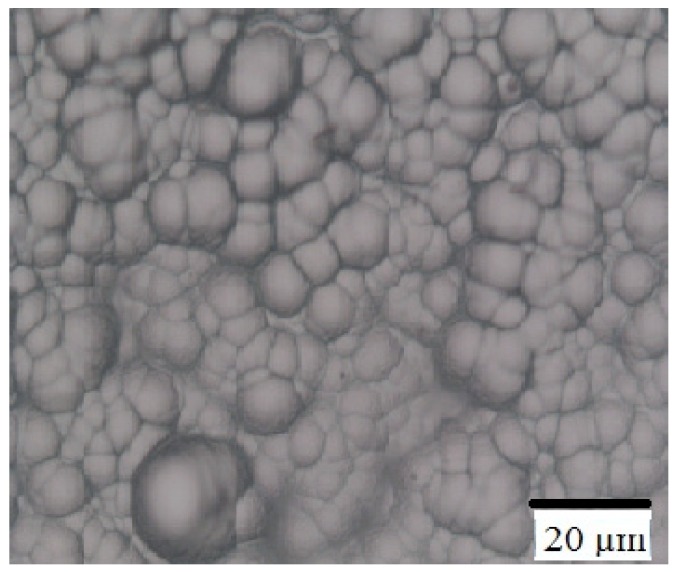
Nomarski photo of grown SiC thin film on graphene/SiO_2_/Si substrate (top view).

Figure 3 shows a typical Nomarski photo (top view) of a grown layer. Since the buffer layer is originally formed by the accumulation of graphene grains, the grown film also shows a grain-like morphology. The grain sizes were consistent with the graphene sizes. The average thickness of the grown layer is determined to be around 2 µm.

**Figure 4:**
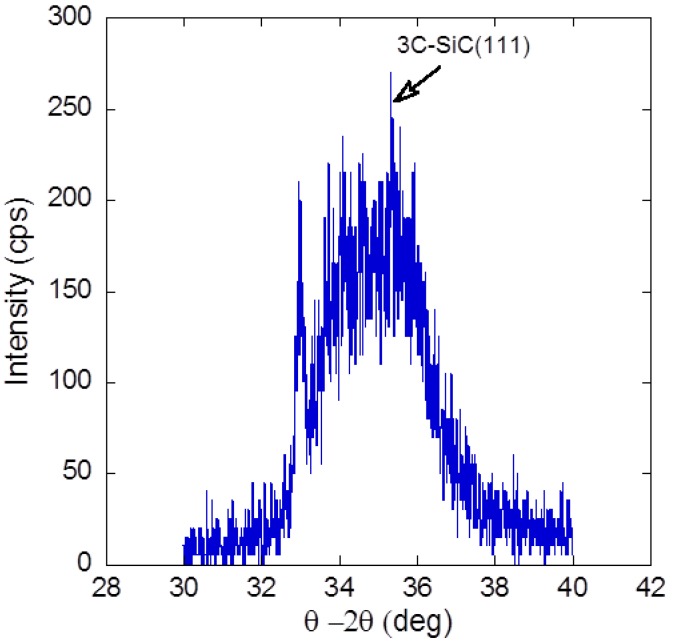
θ-2θ scan of grown SiC thin film.

Figure 4 shows the XRD spectra of θ–2θ scan. Although the spectra is broad and weak, a peak corresponding to 3C-SiC(111) is observed at 35.7° [28]. It is noted that a peak of 3C-SiC(200) at 41.4° was not observed in our measurements [29]. Since only the diffraction peak of 3C-SiC(111) was clearly observed, we speculate that the growth of 3C-SiC on graphene/SiO_2_/Si(100) substrate has been enhanced in (111) domain. These experiments have been repeated, and all samples have exhibited the same orientation of 3C-SiC (111). The full width at half maximum (FWHM) of XRD curve shown in Figure 4 is estimated to be around 2.9°. This broad XRD curve indicates that the crystalline grain size is very small. From the Debye-Scherrer formula, the average size of the crystalline grain is calculated to be around 2.8 nm.

**Figure 5:**
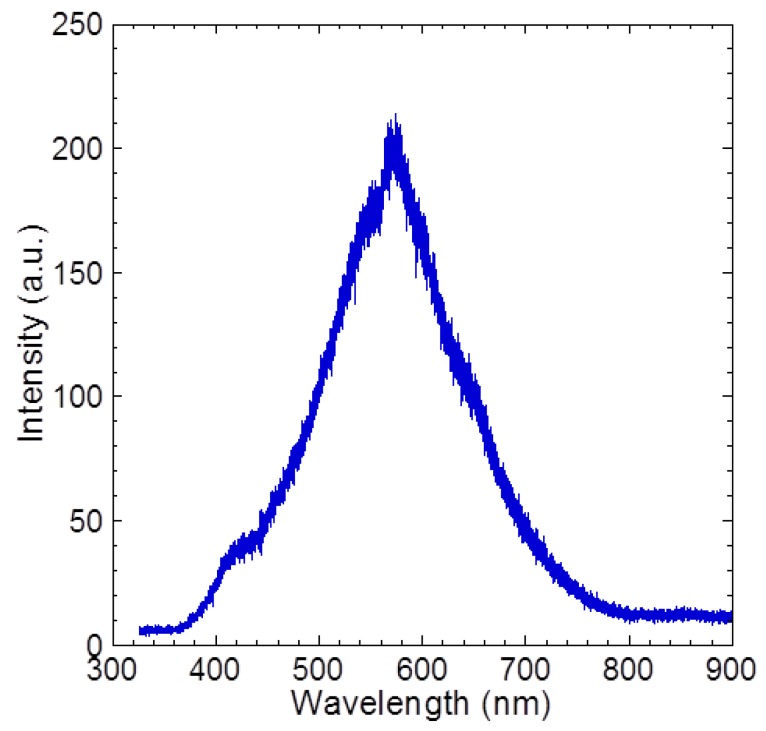
Photoluminescence of grown SiC thin film.

Figure 5 shows the measured room temperature PL spectra of grown SiC film. The curve is symmetric at the wavelength’s peak of 564 nm and this wavelength corresponds to the energy of 2.2 eV. The FWHM is estimated to be around 0.6 eV. The energy bandgap of 3C-SiC was reported to be around 2.2–2.3 eV [30,31]. In this work, the grown SiC films were also polycrystalline; they were the same as those reported SiC films on SiO_2_ [20,21,22], since the polycrystalline single layer graphene flakes were used. However, if the technology to form large area single crystalline graphene is realized and then such a single crystalline graphene structure is applied, it should lead to the realization of a highly oriented single crystalline 3C-SiC (111) continuous thin film. Therefore, it seems to show that graphene is a promising buffer layer to grow single crystalline material structures on amorphous material. Recently, Suemitsu *et al.* reported the graphitization process or the formation of epitaxial graphene on the 3C-SiC (111) surface by an annealing process in an ultrahigh vacuum condition [32]. Therefore, it supports the feasibility of forming 3C-SiC film on graphene since both processes are simply reversed and we speculate the same bonding structures should be formed. However, to elucidate the growth mechanism, detail studies using cross-sectional TEM, low-energy electron diffraction and X-ray photoelectron spectroscopy (XPS), need to be carried out. Several properties, such as activation energy, SiC-graphene binding, destabilization energy, *etc*., also need to be determined, thus opening a lot of interesting works to be explored.

**Figure 6:**
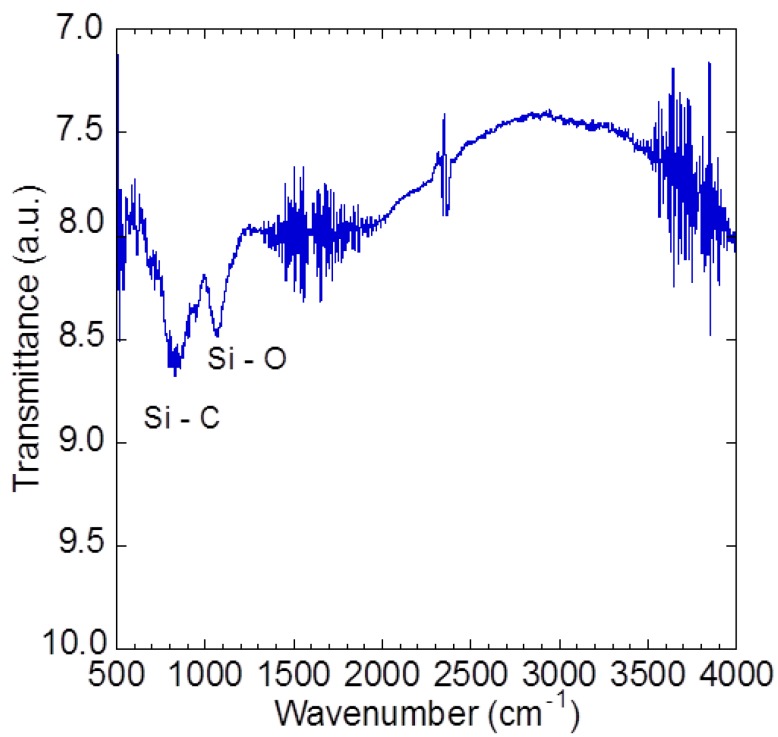
Infrared absorption spectra of grown SiC thin film.

Figure 6 shows the infrared absorption spectra where two absorption bands at 800 cm^−1^ and 1080 cm^−1^ are observed. The peak at around 800 cm^−1^ indicates the transverse optical (TO) phonon of stoichiometric Si-C bond in 3C-SiC [33] while 1080 cm^−1^ indicates the Si-O bond [34]. In general, the Si-C stretching mode for the crystalline state is precisely located at around 796 cm^−1^ for 3C-SiC bulk. In this case, the small variation of absorption band may be due to the size effect of the precipitates/grains. The Si-O absorption band observed in the grown film can be explained by two aspects: (1) Out-diffusion of the oxygen atom from the SiO_2_ substrate; and (2) oxygen doping during the cleaning process. Since the gap between the mesh and substrate was just 30 mm, the surface temperature of the substrate is considerably more than 750 °C. This condition may cause the out-diffusion of oxygen from the SiO_2_ layer into the graphene and grown SiC film. Graphene can also be easily doped by oxygen during the organic cleaning process [35].

The local bonding distortion in an intrinsic SLG can result a π-orbital misalignment [36]. This distortion will weaken the π-bond and increase the chemical reactivity. We speculate that the degradation the quality of SiC thin film is caused by this severe Si–O bonding inside the film.

## 4. Conclusions

In conclusion, these preliminary results suggest that graphene can be used as a promising buffer layer for epitaxial growth of 3C-SiC thin film on insulator at relatively low temperatures. Ideally, graphene should be grown directly on the insulator prior to the growth of SiC film. However, such technology is not realized up to this date. Further experimental works by (1) utilizing large-area SLG; (2) optimizing the cleaning process; (3) optimizing the growth parameters of the present growth method, and (4) utilizing other promising growth methods as well, are expected to realize high quality SiC thin film on insulator. Graphene can not only serve as a promising buffer layer for the growth of semiconductor thin films and nanostructures on insulators in highly oriented single crystalline forms, but it also can be used to fabricate other functional devices by exploiting its superior characteristics towards the realization of advanced ULSIs with multifunctionalities.
